# Chimpanzees show some evidence of selectively acquiring information by using tools, making inferences, and evaluating possible outcomes

**DOI:** 10.1371/journal.pone.0193229

**Published:** 2018-04-11

**Authors:** Bonnie M. Perdue, Theodore A. Evans, Michael J. Beran

**Affiliations:** 1 Department of Psychology, Agnes Scott College, Decatur, Georgia, United States of America; 2 Language Research Center, Georgia State University, Decatur, Georgia, United States of America; 3 Department of Psychology, Georgia State University, Atlanta, Georgia, United States of America; Centre national de la recherche scientifique, FRANCE

## Abstract

Metacognition refers to thinking about one’s thinking or knowing what one knows. Research suggests that this ability is not unique to humans and may be shared with nonhuman animals. In particular, great apes have shown behaviors on a variety of tasks that are suggestive of metacognitive ability. Here we combine a metacognitive task, the information-seeking task, with tool use and variable forms of initial information provided to chimpanzees to explore how informational states impact behavioral responses in these apes. Three chimpanzees were presented with an apparatus that contained five locations where food could be hidden. If they pointed to the correct location, they received the reward, but otherwise they did not. We first replicated several existing findings using this method, and then tested novel hypotheses. The chimpanzees were given different types of information across the experiments. Sometimes, they were shown the location of the food reward. Other times, they were shown only one empty location, which was not useful information. The chimpanzees also could use a tool to search any of those locations before making a selection. Chimpanzees typically used the tool to search out the location of the reward when they could not already know where it was, but they did not use the tool when they already had been given that information. One chimpanzee made inferences about the location of hidden food, even when that food was never shown in that location. The final experiment involved hiding foods of differing preference values, and then presenting the chimpanzees with different initial knowledge states (i.e., where the best food was located, where the less-preferred food was located, or where no food was located). All chimpanzees used the tool when they needed to use it to find the best possible item on that trial, but responded by choosing a location immediately when they did not need the tool. This finding highlights that their behavior was not the result of a simple rule following such as pointing to where any food had been seen.

## Introduction

Metacognition refers to “thinking about thinking” or the monitoring and control processes that occur as part of information processing [1–4]. In humans, metacognition manifests in many forms including seeking additional information when uncertain, gauging the confidence of memory or perception, and reporting such confidence. There is increasing evidence of such abilities in apes and monkeys [5–32], and some evidence of metacognition in non-primate species [33–40]. However, there has been much debate about whether the results reported in the animal metacognition literature should necessarily be interpreted as metacognitive, or whether different explanations are sufficient [41–58]. For this reason, empirical efforts remain important.

One of the most widely used paradigms in the animal metacognition literature is the information-seeking task. In the first use of this paradigm, Call and Carpenter [12] tested children, orangutans and chimpanzees in a spatial memory test where food could be hidden in one of a number of discrete locations. Sometimes subjects were shown the exact location of a prize, but in another condition, subjects were not given that information, although they could look into the hiding locations before making a choice of one of those locations. All three species showed evidence of seeking additional information by looking for the food before trying to obtain it when they had not seen the hiding location. However, when they had seen where the food was hidden, they were more likely to just point to that location. These findings suggested that subjects knew what they did or did not know and responded accordingly. Other species also showed differential looking or pointing responses to trials where information was still needed or was not needed (e.g., lion-tailed macaques [18]; capuchin monkeys [31]; rhesus macaques [59]; [23]; baboons [17]).

One criticism of the information-seeking task is that perhaps animals simply reach toward food but look when they have seen no food, and this could generate some of the earliest reported results without need of assuming animals were aware of their own knowledge states; [41,45]. Subsequent studies addressed some of these concerns by varying the effort needed to search for information or by varying the quality of the rewards, so that different trials offered animals different information states and costs to obtaining more information. In those cases, apes appeared to respond based on what they could know from what they had seen, not based solely on the visibility (or not) of food during a trial. Apes also selectively searched for information depending on the levels of effort required to get that information, depending on the qualities of reward, the relative risks of making errors, and they sometimes even made inferences about where food could be located without having to look [11,19–20].

In a different variation of an information-seeking task, Beran et al. [7] presented language-trained chimpanzees with two potential knowledge states (either knowing the contents of a container or not knowing the contents), but rather than have the chimpanzees directly retrieve food items based on knowledge of locations, the chimpanzees had to correctly name the hidden item in order to obtain it. Thus, criticisms related to beliefs about food location [41] and/or search strategies based on finding food [45] were not applicable. Given all of these extensions and modifications to the original task of Call and Carpenter [12], the value of this information-seeking paradigm remains high with regard to assessing aspects of animal metacognition and studying the underlying mechanisms, constraints on performance, or parallels with human metacognition.

However, there are still only a small number of such studies conducted with apes, and in many of those studies the apes also showed mixed results, which introduces the importance of exploring individual differences and specific factors that underlie performance in animals. We add to those studies with a series of experiments demonstrating many of these same information-seeking phenomena, but with a new aspect to the information-seeking behavior—the integration of tool use. Specifically, we integrated the acceptance or retrieval of a tool as the method by which chimpanzees could acquire information that was not otherwise possible to obtain, because they could make searches of occluded locations. Other studies [19–20] sometimes had apes make operant responses to possible hiding locations with an object (e.g., dowel) as a pointing device, but our integration of tools was designed so that apes could look for information *before* making a choice that then led to food reward or not. And, our tasks required that when apes opted to use a tool, they had to return that tool to an experimenter as a way to indicate readiness to make a response indicating where food was located. The tool was not a means of obtaining the reward, but only a means of gaining information that could be used to gain reward after it was returned. Admittedly, this is an incremental addition of complexity to the information-seeking task rather than a novel approach to assessing information-seeking behavior, but we think it is still important. Behavioral inhibition is necessary for an organism to inhibit a primary response (i.e., reaching to a possible hiding location) in orderto seek additional information before responding. Some past studies had that component in their “look before reaching” measures, although in some cases the primary response could not occur immediately. For example, Marsh and MacDonald [19–20] only allowed their apes in some tasks to make a choice after requiring them to sit through a delay during which they might look into containers or not. Our study, however, was the first that forced chimpanzees to forego the chance to make a response to gain reward if they opted instead to use a tool to seek information. The novelty is in this aspect—in committing to seeking more information, a subject in our studies *gives up* the chance to try to more immediately point out a “found reward” because delay is self-imposed in that aspect of the task.

We integrated the tool component because tool use seen in animals [60] often suggests a connection to monitoring and control processes such as those that are central to most definitions of metacognition [61]. For example, Osvath and Osvath [62] presented chimpanzees and orangutans with a task in which they could choose between an immediately available food item and a tool. The tool was only useful at a future time when, if selected, it then could be used to retrieve a preferred food item. These apes appeared to anticipate the future need of the tool, and selected it accordingly when that led to the best outcome [63]. This provides insight into future oriented cognition in apes, but might also present a useful methodology—in combination with the information-seeking paradigm—for further exploration of metacognition. The idea is that perhaps chimpanzees could decide whether they needed a tool to gather more information *that then allowed them to make a separate response to obtain food*, or whether they already had all of the information necessary to make the correct response to a spatial memory task.

We used this approach of combining tool use and information seeking to conceptually replicate some past results demonstrated with other apes [12,19–20,64] while asking a new question about how chimpanzees might deal with trials in which multiple food types were sometimes presented, and information about food location was varied across those food types. In all of these experiments, chimpanzees had to evaluate what information they had or did not have across varied contexts that included the need to determine the cost of making errors, the possibility that inferential reasoning would allow an individual to skip information-seeking responses even when direct visual information is not available, and the need to carefully attend to object identity and value when deciding whether more information should be obtained or a direct response could be made. Selection of the tool would also commit a chimpanzee to no longer being able to make a direct response to the array of hiding locations, and would thus be a new form of “cost” introduced into this general family of information-seeking tasks.

Chimpanzees first learned that a tool could be used to reveal information needed to later retrieve food. Sometimes chimpanzees needed the tool to gain the information needed to find food, but in other cases the information was provided without requiring use of the tool, although the tool was still available to them. If a chimpanzee had the information needed, whether it was directly shown the food location or if that location could be inferred, we hypothesized that the subjects would not use the tool, but would use it more often for trials for which insufficient information had been provided. The preliminary experiments presented here first refined the methodology and then replicated previous findings [7,12,18–19,31,65] but within the new task component of foregoing immediate opportunities to obtain food for access to a tool to gain information.

In a subsequent test, we assessed whether chimpanzees recognized when they needed to seek more information or instead could make inferences about the location of the hidden item that would negate the need for the tool (and negate the need to delay the chance to choose a hiding location). A final experiment introduced novel conditions in which multiple foods of different quality were hidden in arrays, and food location was no longer the sole piece of information needing to make the best response. Now, chimpanzees needed to know *what was where*, or at least where the best thing was, and this meant that seeing where any food was located was not the same as seeing where the best food was located. The question here was whether chimpanzees would selectively acquire and use the tool when information states were incomplete, even if they had seen where some food item was hidden. This was the strongest test of the required inhibition needed to avoid making a response to the array to instead obtain and use a tool to first seek more information.

## Preliminary task training & general design

Initially we trained the chimpanzees to use the apparatus and the tool within the context of seeing or not seeing the reward item prior to receiving the tool. To do this, we revealed one location that was either baited with the food item (“*food reveal*”) or was empty (“*empty reveal*”). We recorded which, if any, locations were searched with the tool before it was returned. If chimpanzees used the tool more often on the empty reveal trials than the food reveal trials, then this pattern could be consistent with a metacognitive explanation of performance.

### Method

#### Subjects

Three chimpanzees, Lana (female, 45 years old), Sherman (male, 42 years old), and Mercury (male, 29 years old) were tested. All chimpanzees had participated in previous experiments including the metacognition studies noted earlier [7–8]. However, they did not have any previous experience with the tool used in this study. Although they have been exposed to PVC pipe previously, it had never been for the purpose of search behavior being tested here, and the apparatus they engaged in this experiment was new to them. The chimpanzees were tested in their normal living quarters, which included indoor enclosures and outdoor yards. Each chimpanzee was tested while singly housed, but otherwise lived with the other chimpanzees and could engage in normal behavioral routines such as grooming, playing, climbing outdoor towers, and engaging in foraging behavior. They were never food deprived and had access to fruits and vegetables throughout the day and ad libitum access to water. Some food items used during testing were from the regular diet and others were special treats only delivered during testing. Test sessions occurred two to four times per week between 10:30 and 13:00.

This project was reviewed and approved by the IACUC committee of Georgia State University (IACUC #A15018). All guidelines outlined in the Institute of Medicine report for the ethical use of chimpanzees in research were adhered to (and always have been) during all aspects of this research, and the research was in accordance with guidelines for The use of non-human primates in research. Chimpanzees were maintained in social groups throughout the project, with indoor and outdoor access to areas that included enrichment and cognitive challenge.

Chimpanzees were never food or water deprived, and worked on these tasks at their choosing (i.e., they always had the opportunity to disengage from testing at any time). Full time veterinary care was provided by GSU, and there were no restraints or use of anesthesia during this research project.

#### Design and procedure

**Fig 1:**
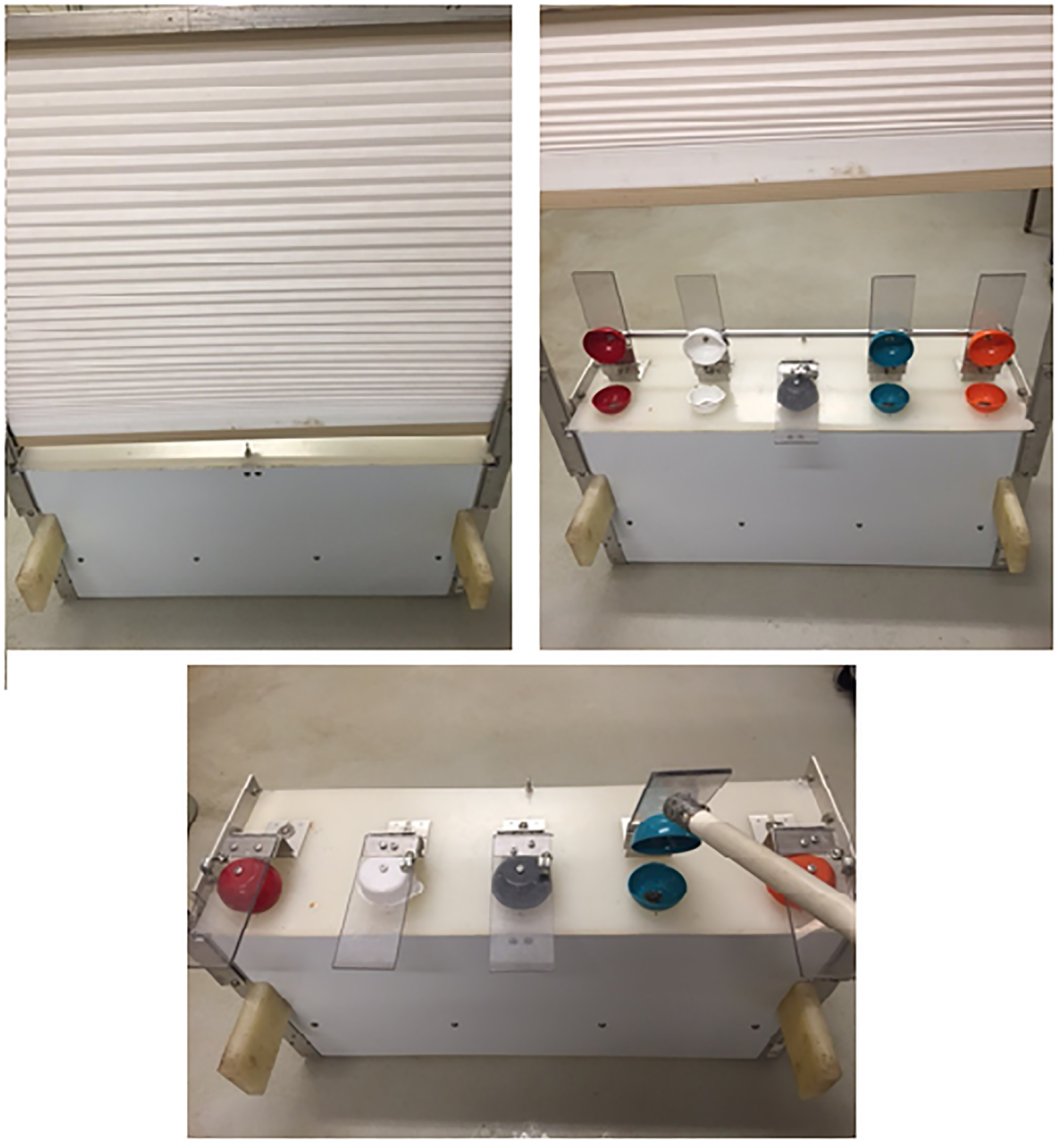
The apparatus. The top left panel shows the chimpanzee’s view of the apparatus when the blind was lowered. The top right panel shows the view when the blind was raised, and the five containers could be seen. Each was covered with a uniquely-colored cover and attached clear lid. Each lid could be raised or lowered using the tool as in the bottom panel. In some cases, as in the top right panel, more than one container could be revealed simultaneously for the chimpanzee to view whether food was hidden in that container or not.

The apparatus consisted of a sliding tray mounted to a rolling test station. The sliding tray contained five containers (see Fig 1) that were each covered with a lid that could be lifted to reveal contents of the container. Lids could be lifted individually or in such a way that more than one could be lifted all at once. This was accomplished by using a bar that could run through two or more of the lids so that they all lifted in unison. There was a screen in front of the tray that could be lifted or pulled down. When the screen was in the down position, the chimpanzees’ view of the tray was completely occluded. When the screen was in the up position, the sliding tray could be pushed within a chimpanzee’s reach so s/he could point to a lid, or it could be pulled back and out of reach of any points, but still close enough that lids could be lifted with the tool. Test sessions were videotaped.

### Baiting

Prior to the onset of the trial, the screen was lowered and Experimenter 1 (E1) stood up and showed the chimpanzee the food item to be hidden by holding it above the test apparatus. E1 then sat behind the screen and proceeded to open and close each of the container lids from left to right. This was to control for auditory and/or temporal cues. A marshmallow was placed into one of the containers during this process. The screen was then lifted so that the chimpanzee could see all covered containers. On some trials, E1 revealed the contents of the baited container by lifting that lid and allowing the chimpanzee to see the marshmallow in the container. This was the *food reveal* condition. On other trials, the chimpanzee was shown an empty container. This was the *empty reveal* condition. Experimenter 2 (E2) did not view this process, and was unaware of where the food item was hidden, unaware of which container had been revealed, and unaware of the condition (*food reveal* or *empty reveal*).

#### Search phase

After the baiting and reveal, the chimpanzee was either directly given the tool or given the choice to take the tool (a piece of PVC pipe) by E2. The tool could be used to lift a lid on any of the containers, but otherwise these lids were not within reach. E2 recorded the order in which the chimpanzee lifted lids with the tool while E1 sat with her head down and her eyes closed throughout the search phase so that she could not give any potential cues to the chimpanzee and could not see what the chimpanzee was doing. Passing the tool out of the enclosure to the experimenter prompted the tray being pushed forward to allow the chimpanzee to make a selection.

#### Selection phase

The chimpanzee then selected one of the locations by pointing to or touching a lid, and E2 verbally announced which location was selected. E1 then looked up and opened the container announced by E2. If there was food in the container, the chimpanzee was given the item to consume. Otherwise, nothing was given. However, subjects saw the correct location when the food was removed on incorrect trials. Then the next trial was prepared.

### Results and discussion

**Fig 2:**
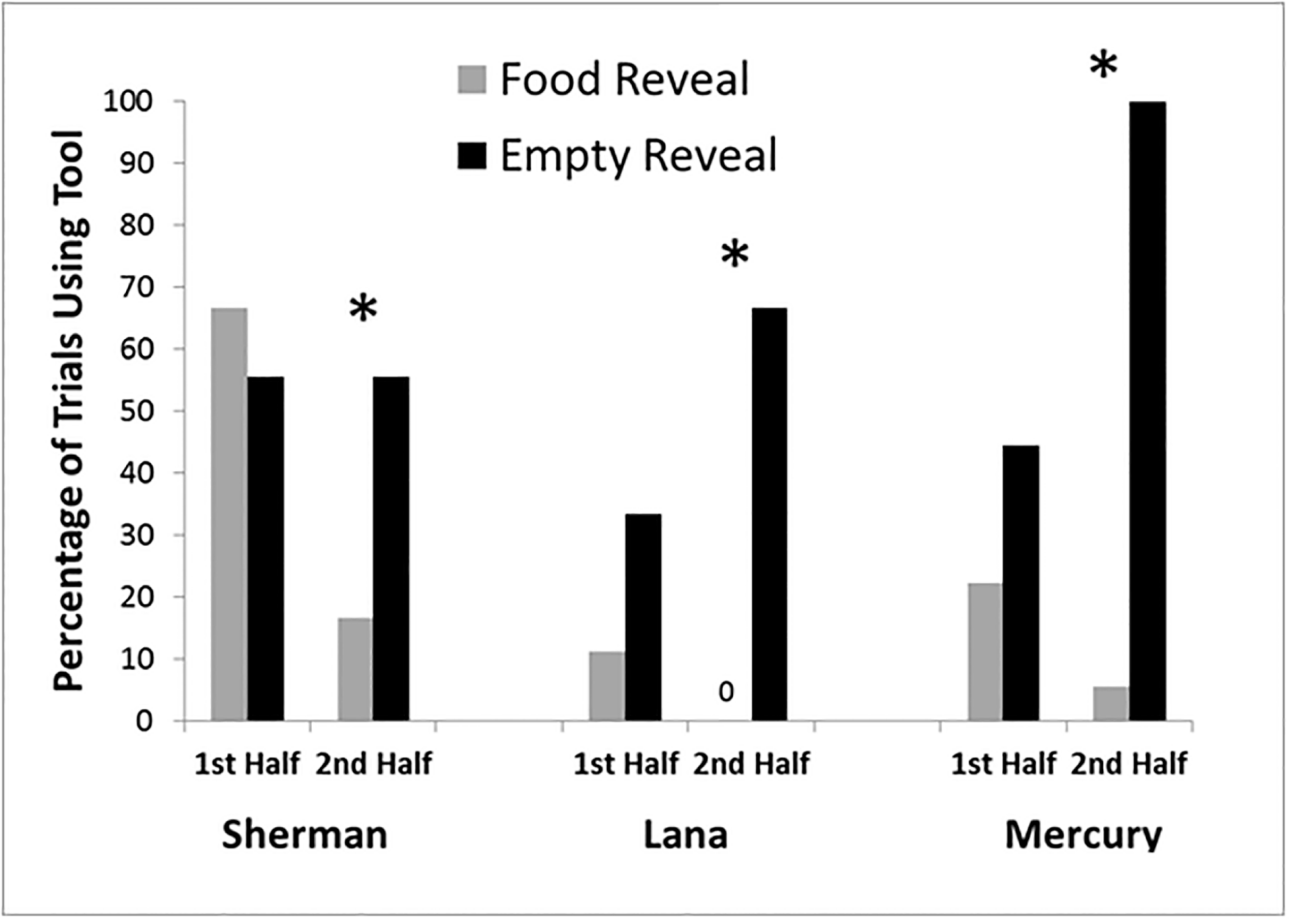
Tool use in Experiment 2. The percentage of trials each chimpanzee used the tool to inspect containers in each condition and for each half of Experiment 2. None of the chimpanzees showed statistically significant differences in use of the tool in the first half of the experiment, but all did so in the second half.

Initially we gave the chimpanzee the tool on every trial, but ultimately found a pattern of over-searching behavior that was not consistent with a metacognitive account (see Table 1). In other words, subjects used the tool to search locations even when they had just been shown the correct location. Their heavy reliance on *redundant searches* was not an indication of good memory monitoring or efficient information-seeking behavior. Rather, it was potentially akin to what Call [11] has described as the “passport effect” in which, when costs are low to confirming information one is already confident one knows, such confirmation occurs frequently (as when a traveler keeps checking a pocket or other easy-to-access location to make sure they brought their passport to the airport). Thus we altered the design so that the chimpanzees had to choose whether or not to take the tool after the baiting event had been completed. This added a small but noticeable addition of effort and time to the trials, with the idea that this might discourage redundant searches. The use of adjusted effort in association with information seeking responses contributed to the successful outcome of Hampton et al.’s [59] study with rhesus macaques, and we expected it to do the same here. Now the chimpanzees were given the choice between obtaining the tool and choosing from the tray immediately. After baiting was completed and one container was shown, E1 said “sliding forward” and slid the tray within reach of the chimpanzee. At the same time, E2 (who again was unaware of the location of the food and unaware of what information the chimpanzee had been given) pushed the tool partially into the enclosure through the mesh (while continuing to hold onto one end of the tool) and approximately 1 meter to the side of the chimpanzees. If a chimpanzee touched the tray by indicating one of the containers, the tool was withdrawn and the selected container was opened. If it contained the food item, it was given to the subject. If a chimpanzee reached over and took the tool, the tray was withdrawn to a distance that it could only be reached with the tool and the subject was allowed to search locations indefinitely. When the tool was passed to the experimenter, the subject was able to choose one of the containers. We gave each chimpanzee 12 sessions of six trials each. For the last 36 trials, the chimpanzees obtained the tool to search significantly more often in the *empty reveal* condition than the *food reveal* condition; Sherman Χ^2^ (1, N = 36) = 5.90, *p* = .02; Lana, Χ^2^ (1, N = 36) = 18.00, *p* < .001; Mercury Χ^2^ (1, N = 36) = 22.21, *p* < .001 (see Fig 2) and were highly accurate in pointing to the correct location after returning the tool to E2. In trials in which chimpanzees selected the tool first, Sherman was correct on 25 of 27 trials (92.6%), and Lana (15 of 15) and Mercury (25 of 25) were correct on 100% of trials.

**Table 1:** Chimpanzee search behavior during preliminary training.

	Food Reveal		Empty Reveal		
	Redundant Searches	Exploratory Searches	Correct Location Searches	Redundant Searches	Exploratory Searches
Sherman	22	2	9	4	13
Lana	22	0	8	6	12
Mercury	19	2	5	9	13

Note. *Redundant searches* were those in which the chimpanzee searched the location that had been revealed. In the *food reveal* condition this was the location that held the food reward, and in the *empty reveal* condition this was an empty location. *Exploratory searches* were any searches to containers other than the one that had been revealed. *Correct location searches* in the empty reveal condition were trials in which the chimpanzees found the food, by chance (20%), on the first search. Sherman (34%), Lana (31%), and Mercury (19%) did not differ from chance in these searches, all *p* > .05, binomial test.

Experience with the task was necessary before this pattern of responding emerged as subjects may not have immediately understood the value of returning the tool immediately (see Fig 2), but it is important to note that no specific element of the environment (e.g., the food item, the apparatus, the baiting procedure, etc.) could tell the chimpanzee whether or not they had the information needed to locate the food item on any given trial. These results suggest that, with some experience, this modified version of the information-seeking task provides a meaningful way to investigate metacognition with findings similar to previous work. However, one of the main criticisms of this paradigm remains plausible at this point—the chimpanzees may have been following a rule that does not require metacognitive monitoring. For example, a rule such as “reach toward food when it is seen and search for food (with the tool) when nothing is seen” might have created the same pattern of behavior. One potential way to avoid this alternative explanation is to design the task in such a way that the correct location is actually one in which food has never been seen. Thus, we incorporated inference into the design to explore the possibility that subjects might be able to monitor their knowledge and respond to a location not based on a visual cue or trace, but on an inference that food should be there [12,18–19,31]. If the chimpanzees avoided retrieving the tool and pointed directly to the location that had to hold the food (i.e., because they saw all other locations as being empty), this would demonstrate that a rule-based strategy such as the one just outlined could not be the sole explanation for tool use and responding by the chimpanzees.

## Inference and information-seeking

Other studies have examined inference in a metacognitive context [12,18,31,65]. For example, Marsh and MacDonald [19] tested whether orangutans could infer the location of a hidden food item and monitor such knowledge of food location to efficiently solve a task. The orangutans produced mixed results. If chimpanzees could make such inferences in our tool-based task, this would indicate that they were not using a rule grounded in the presentation of food. Instead it would indicate that they were using their knowledge of where food was located to retrieve the tool or not. If chimpanzees responded to the inference-based information in a similar manner to actual visual information about food location, this pattern of responding would support a metacognitive explanation of responding.

### Method

#### Subjects

The same chimpanzees were tested.

#### Design and procedure

The procedure was the same as the general procedure described above with the exception of the reveal phase. The baiting still occurred behind the screen, all lids were touched and lifted, and only one container was baited with food. For this experiment, there were three conditions: 1) only the baited container was revealed (*food reveal* condition), 2) only one empty container was revealed (*empty reveal* condition) (these first two trial types are the same as in the earlier phases), or 3) we simultaneously revealed four empty locations (see Fig 1, top right panel) (*inference reveal* condition). In this final condition, the chimpanzees could not directly see the baited container with a food item in it, but they potentially could infer the location of the food item because all of the other locations were shown to be empty.

Each test session consisted of eight trials—two *food reveal*, three *empty reveal*, and three *inference reveal* trials in which four empty locations were revealed. The location of the food item and the order of these trials was randomized as in earlier phases. Each chimpanzee completed five test sessions.

### Results and discussion

**Fig 3:**
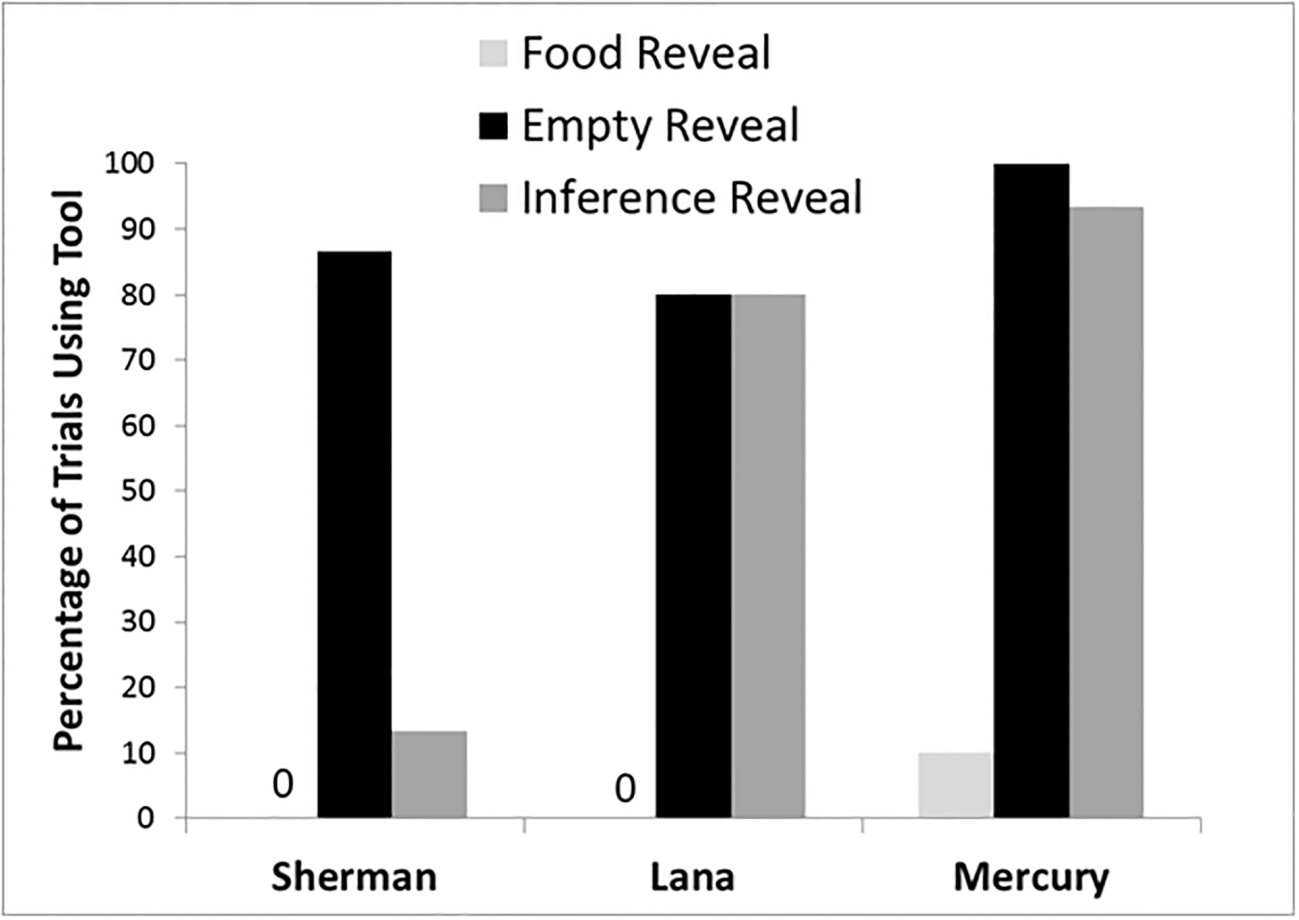
Tool use in Experiment 3. The percentage of trials each chimpanzee used the tool to inspect containers in each condition in Experiment 3. A pattern indicative of inferential reasoning would be apparent if use of the tool in the *inference reveal* condition was as low as the *food reveal* condition, versus the expected high use of the tool in the *empty reveal* condition. Only Sherman showed this pattern indicative of inferential reasoning.

The chimpanzees were highly proficient in choosing the correct container after returning the tool to the experimenter (Sherman– 14/15 trials correct; Lana– 24/24 trials correct; Mercury– 27/30 trials correct). The same was true on trials in which they saw the baited container and did not use the tool (Sherman– 9/10 trials correct; Lana– 10/10 trials correct; Mercury– 9/9 trials correct; note that Mercury opted to use the tool on one trial in which the baited container was shown). Fig 3 presents the percentage of trials in which each chimpanzee retrieved the tool and searched locations before returning the tool and choosing a container. Sherman demonstrated a pattern that indicated he was using inferences about the location of food. He selected the tool more often when he was shown a single empty container than when he was shown four empty containers (and could infer the correct location) or was shown the baited container. Thus, our hypothesis was supported for one individual.

Lana and Mercury selected the tool more on *empty reveal* trials than *food reveal* trials. However, on *inference reveal* trials, they also retrieved the tool as often on as on *empty reveal* trials. These two chimpanzees differed somewhat in what they did with the tool on inference trials. This pattern of mixed results among subjects supports the findings of Marsh and McDonald [19] with orangutans.

Lana seemingly did not learn much from what was shown in the inference trials, as she first searched the only container not revealed by the experimenter on 6 of 12 trials. She otherwise searched containers fairly randomly on those trials even though she had just seen those containers as being empty. Mercury, however, was more proficient. He searched only the container that had not been revealed on 9 of 14 trials, and in 4 of the other 5 trials his second search was in that location, suggesting he focused his searches on the container he had not seen revealed in this condition.

Sherman seldom selected the tool on trials in which he could infer the location of the food even though he had never directly seen that food item. Thus, Sherman was not using a rule such as “retrieve the tool when no food was shown, but point to a container when food was shown.” The other chimpanzees continued to show strong performance in terms of obtaining the food, and in terms of only using the tool when they were not shown exactly where the food item was located.

## Effort and information-seeking

This experiment was designed to increase the cost of tool retrieval in an effort to eliminate the convenience of using that tool, to see whether this changed the behavior of Lana and Mercury while continuing to indicate more sophisticated responding from Sherman. In an effort to address the issue of *redundant searching*, we added a greater cost to selecting the tool in the present experiment. We did this by requiring movement to an adjacent enclosure to select the tool if the chimpanzees wanted to use it to search the array. This manipulation would help to clarify whether the chimpanzees perceived that it was necessary to use the tool even when they had the information they needed to forego tool use, or whether the tool often was selected out of convenience because it was presented next to the test apparatus with the well locations (i.e., a *passport effect*). If the chimpanzees continued to select the tool more on the empty reveal trials, even though more effort was required, this would provide additional support that this pattern of responding is metacognitive.

### Method

#### Subjects

The same chimpanzees were tested.

#### Design and procedure

The procedure was the same as the general procedure described above with the exception of the location of E2 and the tool at the beginning of the search phase. Now E2 was in front of an adjacent enclosure that connected to the enclosure with the apparatus and the chimpanzee. Because of the mesh nature of the enclosures, E2 and the tool were still visible to the subject even in the new location. Thus, the key difference for this experiment was that the tool was now located a greater distance away, and this required the chimpanzees to move away from the tray to get the tool. As before, if the chimpanzees touched the tray, the tool was withdrawn. If they took the tool, the tray was withdrawn from reach but the containers on the tray could be searched with the tool.

Each test session consisted of eight trials—four *food reveal* trials and four *empty reveal*. Baited container location and trial order were randomized. Each chimpanzee completed 5 test sessions.

### Results and discussion

The chimpanzees showed highly proficient choice behavior with regard to when they retrieved the tool and which location they selected from the array. None of the chimpanzees retrieved the tool on trials where they were shown the location of the hidden item. Lana and Sherman retrieved the tool on 75% of the *empty reveal* trials, and Mercury retrieved the tool on 95% of those trials. For all chimpanzees, this differential retrieval of the tool was statistically significant; Sherman Χ^2^ (1, N = 20) = 24.00, *p* < 001; Lana, Χ^2^ (1, N = 20) = 24.00, *p* = < .001; Mercury Χ^2^ (1, N = 20) = 36.19, *p* < .001. As in all previous experiments, selection of a container after returning the tool was highly accurate in terms of where the food item was hidden (Sherman, 93% correct; Lana, 100% correct; Mercury, 94% correct).

These results supported our hypothesis and indicated that, when costs for tool use were increased, the chimpanzees showed a clear pattern of responding based on knowledge states. Of course, additional experience with the task likely also contributed to the increased efficiency of responding by the chimpanzees.

## Complex informational contexts and information-seeking

The pattern of performance in the increased effort condition was suggestive of a metacognitive pattern of performance and replicated findings from other studies [11,19–20,59] using a different methodology. Given that the viability of the technique had been well-established, we next tested whether the chimpanzees could monitor more complex informational contexts. We manipulated the quality and location of single or multiple food rewards to see if chimpanzees continued to optimally monitor their knowledge states and seek information when needed. Again, this study is motivated by the important criticism of the information-seeking task that subjects could respond based on cues from the food itself. To test the possibility that subjects would always respond to the sight of a given food item by choosing the location of that food, we manipulated the availability of other options on trials and what information was made available. Could subjects flexibly respond to the sight of a relatively lower preference item? In other words, would subjects engage in searching behavior after seeing the location of a hidden food item? This pattern (searching after seeing food) is the direct opposite of the traditional pattern of responding in this paradigm (including in our earlier Effort task) and would provide evidence against the notion that basic food searching rules can account for performance on information-seeking tasks.

On each trial, the chimpanzees were shown that only a lower preference item (grape) would be hidden or that a lower preference item (grape) *and* a higher preference item (marshmallow) would be hidden. The preference for marshmallow over grape was confirmed for all chimpanzees before this phase began. Then, out of view of the chimpanzees, either one container was baited with a lower preference food item or two containers were baited, one with a higher preference item and one with a lower preference item. After baiting, the chimpanzees were shown one container that either contained the lower preference item, the higher preference item, or was empty. We made the following predictions that would be in line with a metacognitive account:

If both food types are baited: In the *empty reveal* condition, the chimpanzees should use the tool and search until the higher preference item is located. In the *food reveal—lower preference* condition, in which subjects were shown the lower preference item, the chimpanzees should use the tool and search until the higher preference item is located. In the *food reveal—higher preference* condition in which subjects were shown the higher preference item, the chimpanzees do not need the tool and should immediately select the container with that higher preference item.

If only a low preference food is baited: In the *empty reveal* condition, the chimpanzees should use the tool to search until the lower preference food is located. In the *food reveal—lower preference* condition, the chimpanzees should not use the tool and immediately select the container with the lower preference food.

### Method

#### Subjects

The same three chimpanzees were tested.

#### Design and procedure

The procedure was the same as the general procedure described above with the exception that sometimes only a lower preference item (grape) would be baited, and sometimes a lower and a higher preference item would be baited. Subjects were shown the items before the baiting occurred. After baiting, E1 revealed one container that held a higher preference item, a lower preference item, or was empty.

Each test session consisted of 10 trials. There were five trial types (followed by a description of the behavior that would support a metacognitive account):

1. Lower preference item presented only, and it was revealed.
  - Choose immediately, do not select tool.
2. Lower preference item presented, but an empty container was revealed.
  - Choose tool, search until grape is located.
3. Lower and higher preference items presented, but only the container with the lower preference item was revealed.
  - Choose tool, search until marshmallow is located.
4. Lower and higher preference items presented, but only the container with the higher preference item was revealed.
  - Choose immediately, do not select tool.
5. Lower and higher preference items presented, but only an empty container was revealed.
  - Choose tool, search until marshmallow is located (even if grape is located, continue searching until the higher preference item is found).

These trial types were presented in random order. A total of 10 trials of each of these five types were presented across six test sessions.

### Results and discussion

**Fig 4:**
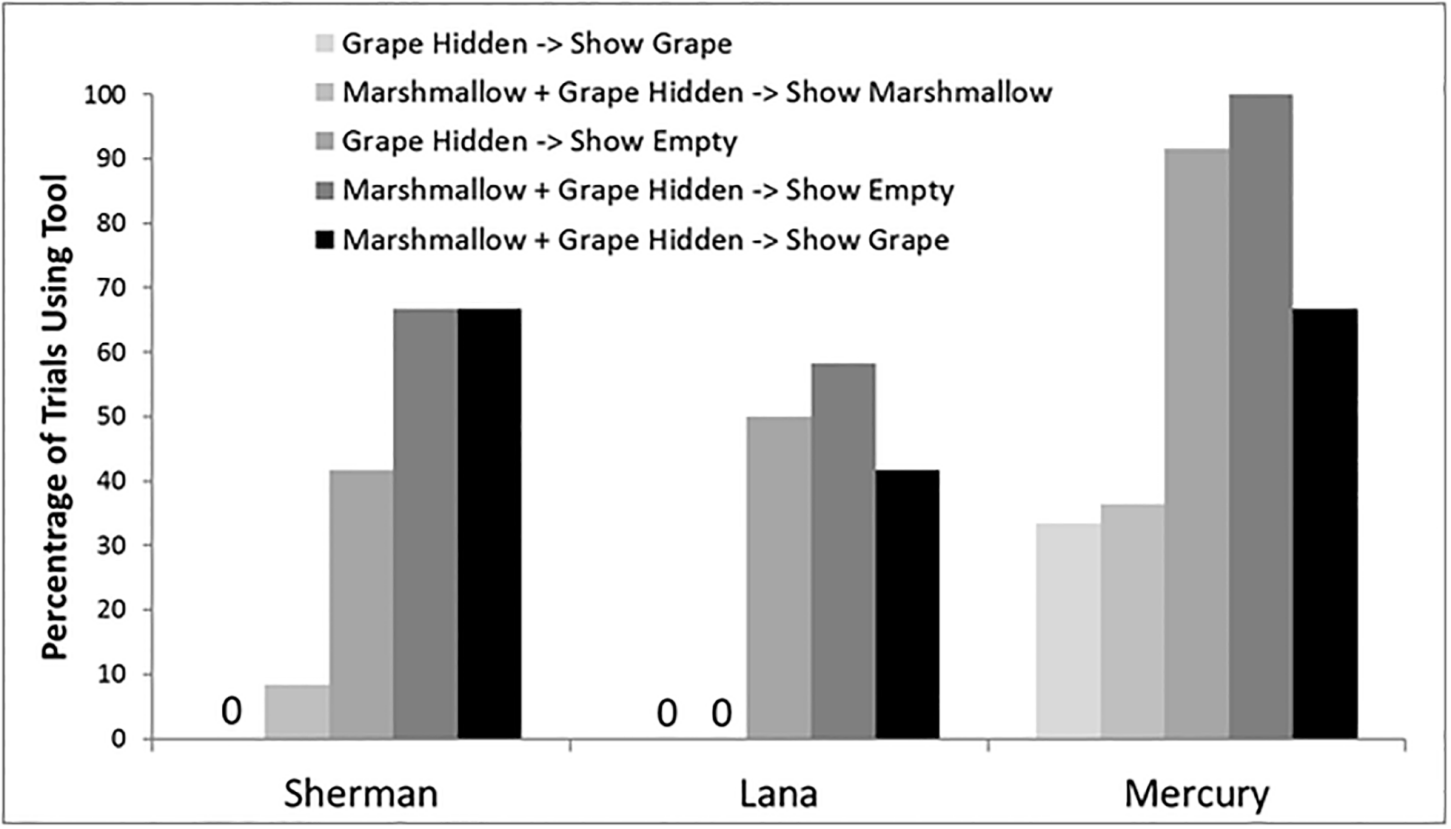
Tool use in Experiment 5. The percentage of trials on which each chimpanzee used the tool to inspect containers in each of the five test conditions in Experiment 5. The two lightest colored bars are trial types in which one would expect little or no use of the tool given that all information about where the best item on that trial was located was already provided. For the three darkest bars, the chimpanzees could not know where the best food item on that trial was located without using the tool.

Fig 4 presents the percentage of trials in which the tool was selected for the five trial types. One thing that was apparent was that the chimpanzees tended to use the tool less often on trials in this condition, when they should have, compared to the earlier tests (e.g., Fig 3). This likely was the result of the more complicated procedure with new trials types, and potentially more information for the chimpanzees to remember. Despite this, all chimpanzees showed the pattern expected in terms of relative likelihood of selecting the tool if the chimpanzees monitored what they were shown, searched with the tool until they found the best food item on that trial (or were already shown the location of that best item), and then made a selection.

The chimpanzees were less likely to select the tool when shown the location of the marshmallow when both a marshmallow and a grape were hidden (0% of trials for Sherman, 8% of trials for Lana, 36% of trials for Mercury), and on all of these trials the chimpanzees pointed to the location of the marshmallow. More importantly, the chimpanzees typically rejected the tool when shown that a grape was the only item that could be received on a trial and then shown the location of the grape. On those trials, Lana and Sherman selected the tool on 0% of the trials, and Mercury selected the tool on 16% of the trials. The chimpanzees also were highly proficient at choosing the location of the grape after rejecting the tool (Lana and Sherman– 100% correct; Mercury– 87.5% correct). Comparing the proportion of trials selecting the tool for these two trial types showed no differences for any of the chimpanzees, *p* > .50, Fisher’s exact test. The chimpanzees seemingly knew what the best item was on a trial, and when shown that item, they typically rejected the tool and pointed immediately to the correct well.

Another important comparison was between trials in which only a grape was hidden and was revealed by E1 versus trials in which a grape was revealed by E1 but a marshmallow also had been hidden (see Fig 4). The latter trials should have elicited more tool retrieval, and they did for all three chimpanzees, although this was statistically significant only for two of the chimpanzees: Fisher’s exact test—Sherman *p* = .002, Lana *p* = .04, Mercury *p* = .22.

Finally, the chimpanzees remained highly proficient at searching only until the best item possible on that trial had been found, at which point they returned the tool and made a location response. Sherman and Lana did this on 100% of the trials in which they selected the tool, and Mercury did so on 81% of the trials in which he selected the tool.

These results indicate that the chimpanzees were sometimes selective about when they used the tool and how they used the tool. The chimpanzees did not always search for a higher preference food. They did so mostly when such a food was available on that trial. Thus, they were adaptive in their tool use and container-selection strategies according to what was available and according to what they knew or did not know about where specific items were located. However, it was also true that they did not use the tool as often as it was required to obtain the best food item. For example, Lana used the tool when it was needed on only about half of the trials, suggesting that this experiment introduced a level of subjective difficulty in assessing what was needed that went beyond that of Experiment 3, where only one food item was presented. These data may therefore reflect a level of metacognitive monitoring in these chimpanzees, but one that was either inconsistently experienced or inconsistently applied in terms of the use of the tool and the search behavior of these chimpanzees. Also, importantly, these findings provide further support against the criticism that responding on info-seeking tasks might be explained by food searching behavior, because in the current work, subjects sometimes continued searching even after food had been seen. This response is not consistent with this alternative account.

## General discussion

Three chimpanzees observed as an experimenter provided them with different kinds of information across a series of experiments investigating how they might use a tool—not to obtain food directly—but to obtain information that could be used at a later time. In some cases, they monitored the relation between the information they received and the likelihood of that information leading to a hiding location for a food item. In general, when they saw where the food was hidden, they immediately acted to make a response to indicate that location. When the chimpanzees did not see where the food was located, they retrieved a tool that could serve to provide additional information, until they found the location of the food item, at which point they returned the tool and made a response to indicate that location. This structured behavior, and differential use/avoidance of the tool across varied task settings, indicates another form of cognitive control and information-seeking behavior that would seem to reflect a metacognitive mode of information processing. One persistent criticism of the information-seeking paradigm is that subjects might respond in a putatively metacognitive manner based on cues from the food itself. In our series of studies, we found support that chimpanzees can choose a location in which food has never been seen and they can avoid choosing locations where food has been seen if a better option remains elsewhere.

In our preliminary training, we found that it was important to give the chimpanzees *choice* of whether to take the tool or not, and this manipulation seemed to clarify the task structure for them, although it took some time. At the beginning of the experiment, two of three chimpanzees already showed a non-significant but differential pattern of choosing the tool or pointing to a location as a function of what was shown to them during the baiting phase. By the second half of the experiment, and then through nearly all subsequent experiments of this study, the chimpanzees used the tool when they did not see the food item, and immediately opted to point to a container on trials where they had.

The next step was to see whether the chimpanzees could experience new kinds of informational contexts and transfer their tool-use or non-tool-use choices appropriately. Thus, all subsequent experiments were designed to require flexible responses in deciding whether to retrieve the tool or not based on what the chimpanzees could know about the location of the hidden food, and eventually also about the different kinds of hidden foods.

We assessed whether the pattern of tool retrieval and use seen in our preliminary task would hold when knowledge of food location came indirectly, through the use of inferences about where food had to be. In that situation, the chimpanzees were shown everywhere that the reward *was not hidden*. This could have cued them as to where food had to be, thereby negating the need to retrieve and use the tool. For one chimpanzee, Sherman, this was exactly how he performed the task, using what he learned about where food was not, to immediately point to where food had to be. Although the other two chimpanzees did not make this “inferential leap”, they continued to selectively choose the tool (or not) based on what they saw directly (an empty container or a baited container). Also, Mercury seemed to selectively search the non-revealed container on the inference trials, suggesting that he may have been confirming the location of the food item in the one location where it likely had to be.

Although this is not the first demonstration of this kind, it is an important finding for multiple reasons: first, Sherman’s success indicates that it is within the capabilities of a non-human animal to modulate information-seeking responses that require using tools on the basis of inference in a novel paradigm. Other animals also have shown responding through exclusion to varying degrees, including many primate species [12,18,19–20,31,65–74], but the important issue here is that Sherman suspended his normal pattern of retrieving a tool when no food was seen from Experiment 2 because he could make that inference. Thus, the second important aspect of this outcome is that Sherman was not using a rule such as “retrieve the tool when I do not see food”, highlighting that such tool-retrieval was not under the external stimulus control of food visibility on a trial. This result adds to that of Marsh and MacDonald [19] showing that some apes can use inferences when given information-seeking tasks. However, Lana and Mercury did not show this pattern. Even though they were good at obtaining the reward, they appeared to rely more heavily on visibility as a cue to tool retrieval or not, which is consistent with a non-metacognitive account. This may highlight a limitation on the group-level flexibility of information-seeking responses that are modulated based on context, a point also reported by Marsh and MacDonald [19].

It is possible that there was some degree of interference from the need to use tools generally to the ability of Lana and Mercury to make inferences, especially given past research that indicated that great apes can make such inferences in non-tool contexts. This might be a form of “cognitive load” interference that disrupted the use of inferences when the chimpanzees had to also concentrate on the role of the tool in the task. Such effects of cognitive load have been reported in other studies with nonhuman primates [26,31,75]. However, it is also possible—and perhaps more likely—that in this experiment Lana and Mercury simply continued using the effective rule from Experiment 2 in which they retrieved the tool when not seeing any food and pointed directly toward food when they saw. This response pattern has been highlighted before as a possible concern for this information-seeking task, where the argument is that rules such as these do not require metacognitive monitoring [41,45,59], and here we also suggest that Lana and Mercury likely adopted a non-metacognitive strategy.

When we increased the amount of effort to obtain the tool, all chimpanzees showed more selective tool use behavior This outcome nicely supports the idea of the *passport effect* [11–12], in the sense that more effort to confirm information one already has tends to decrease the likelihood that one will make such redundant information searches. As in other work with nonhuman primates, varying effort level to obtain information seems to impact information-seeking behavior [11,20,76]. And, it did so quickly, given that we presented only small numbers of trials in each of our later experiments, to minimize the chances that the chimpanzees could learn how to respond to differing trial types through mass experience with those trial types. Thus, the results of the Effort Condition added another layer of generalization of the tool-retrieval or non-retrieval behaviors as those best met the information that chimpanzees had from the trial presentation. Again, a simple rule could not be followed in this task, but the optimal response depended on what information the subject had access to. Further, this manipulation allowed Mercury and Lana to show clearer evidence of the expected patterns of responding.

Finally, we added a level of complexity with the introduction of two food types, one or both of which could be presented on a given trial. Now, chimpanzees had to monitor what would be hidden on a trial, and then what they were shown, to determine what the best available food item would be on a given trial. This introduced the aspect of reward value, and how it might impact tool retrieval and use. An important practical implication of this manipulation is that subjects were now seeing a food item on some trials, but had to inhibit responding to it when other items were available. This further strengthens a metacognitive account of performance on the information-seeking task because in this case, the subject sees the location of a food item, but it is not always ideal to respond to that location if the subject can monitor information about other options available in the environment. When the best available item was shown in a container, typically the chimpanzees immediately pointed to that container, even when the best available item on that trial was not the more preferred of the two items used across trials. When the best available item was not revealed, the chimpanzees used to tool until they found that item, again when the item itself was the more preferred and when it was the less preferred of the pair. This was an important outcome because, as in Experiment 4 (and Experiment 3 for Sherman) it reflected that chimpanzees were not using simple rules such as “point where you see the most preferred reward” but instead were calibrating their tool-retrieval and response behaviors trial-by-trial on the basis of what was known on that trial and on the basis of what could be obtained on that trial. The chimpanzees did not structure their tool use toward searches for the best food *in the session* but rather only for the best available item they could get *on that trial*. This was the strongest evidence in this study for flexible information-seeking behavior that included remembering what could be obtained, knowing what had been revealed, and then using the tool (when necessary) to find the location of whatever item was the best outcome on that particular trial.

This overall pattern of information-seeking behavior also adds another level to the argument for metacognitive processes in chimpanzees. These specific chimpanzees now have shown selective information-gathering behavior when asked to name hidden food items [7] and flexible and appropriate confidence movements reflecting accurate degrees of certainty about memory strength and knowledge [8]. In each of those cases, the methodology prevented any role for response competition [59] because in Beran et al. [7] the response was not toward or away from food, but was about naming food items at a distance from where they were located, and in Beran et al. [6] the task involved retrieving expected rewards that were delivered across a variety of computerized memory tasks that offered no competition for responding except as might accompany the chimpanzees’ sense of whether reward was forthcoming due to correct responding or not. The present experiments certainly allow for a greater chance that these behaviors could be explained on the basis of relative response competition strength from seeing (or not) food items given that food retrieval from various spatial locations is the basis of the task. However, in at least Sherman’s case, this argument does not hold for those trials where he did not see food, but still responded to a location through use of exclusion, and in the final experiment the use of more than one food type at least disrupted responding solely on the basis of response competition predicated on food visibility. Sherman’s behavior in the Inference Condition also indicates that in at least one case a chimpanzee responded on the basis of other than generalized search [41,54] given that he did not search until seeing food, but instead inferred its location and responded appropriately. That he was the only chimpanzee to do this, however, does highlight that this is not an easy condition, and that perhaps multiple strategies are engaged by chimpanzees in these kinds of tests, some of which rely less on metacognitive experiences and more on learning-based approaches to engaging the task.

More broadly, though, the evidence is mounting that apes show flexible information-seeking behaviors in a variety of similar tests [11–12,19–20] as do some monkeys [17–18,23,31,59]. Although no single test may bear the burden of proving or even strongly indicating metacognition in nonhuman animals, the growing database of successes in varied tests such as these does suggest a strong capacity in great apes (and perhaps other species) for the monitoring and control processes that are central to the workings of a metacognitive system [1,77]. Of course, these processes need not be accompanied by the self-reflective, conscious awareness of monitoring one’s own thoughts that adult humans (sometimes) employ, although they might. Ultimately, they do not have to share those phenomenological qualities to indicate controlled cognition within species other than humans, and to indicate perhaps how the early precursors of humans’ full-fledged metacognitive abilities would have operated.
